# Associations Between the Purinergic Receptor P2X7 and Leprosy Disease

**DOI:** 10.3389/fgene.2021.730991

**Published:** 2021-11-02

**Authors:** Rebeka da Conceição Souza, Thaís Louvain de Souza, Cristina dos Santos Ferreira, Letícia Silva Nascimento, Edilbert Pellegrini Nahn, Alba Lucínia Peixoto-Rangel

**Affiliations:** ^1^Laboratório de Biologia do Reconhecer, Centro de Biociências e Biotecnologia, Universidade Estadual do Norte Fluminense Darcy Ribeiro, Campos dos Goytacazes, Brazil; ^2^Faculdade de Medicina de Campos, Campos dos Goytacazes, Brazil; ^3^Núcleo de Diagnóstico e Investigação Molecular, Laboratório de Biotecnologia, Centro de Biociências e Biotecnologia, Universidade Estadual do Norte Fluminense Darcy Ribeiro, Campos dos Goytacazes, Brazil; ^4^Laboratório de Bioinformática, Laboratório Nacional de Computação Científica, Petrópolis, Brazil

**Keywords:** leprosy, *P2RX7*, c.1513A>C, rs3751143, c.1068A>G, rs1718119, RNA-seq

## Abstract

Leprosy is an infectious disease still highly prevalent in Brazil, having been detected around 27,863 new cases in 2019. Exposure to *Mycobacterium leprae* may not be sufficient to trigger the disease, which seems to be influenced by host immunogenetics to determine resistance or susceptibility. The purinergic receptor P2X7 plays a crucial role in immunity, inflammation, neurological function, bone homeostasis, and neoplasia and is associated with several infectious and non-infectious diseases. Here, we first compare the *P2RX7* expression in RNA-seq experiments from 16 leprosy cases and 16 healthy controls to establish the magnitude of allele-specific expression for single-nucleotide polymorphisms of the gene *P2RX7* and to determine the level of gene expression in healthy and diseased skin. In addition, we also evaluated the association of two *P2RX7* single-nucleotide polymorphisms (c.1513A>C/rs3751143 and c.1068A>G/rs1718119) with leprosy risk. The expression of *P2RX7* was found significantly upregulated at macrophage cells from leprosy patients compared with healthy controls, mainly in macrophages from lepromatous patients. Significant risk for leprosy disease was associated with loss function of rs3751143 homozygous mutant CC [CC vs. AA: *p* = 0.001; odds ratio (OR) = 1.676, 95% CI = 1.251–2.247] but not with heterozygous AC (AC vs. AA: *p* = 0.001; OR = 1.429, 95% CI = 1.260–1.621). Contrary, the polymorphic A allele from the gain function of rs1718119 was associated with protection for the development of leprosy, as observed in the dominant model (AA + AG × GG *p* = 0.0028; OR = 0.03516; CI = 0.1801–0.6864). So, our results suggest that the functional P2X7 purinergic receptor may exert a key role in the *Mycobacterium* death inside macrophages and inflammatory response, which is necessary to control the disease.

## Introduction

Leprosy, caused by *Mycobacterium leprae*, is a disease that affects the mucosa, skin, and peripheral nerves. It is highly prevalent in Brazil and represents a public health problem, with 27,863 new cases in 2019. The country ranks second in the global incidence of leprosy, behind India [World Health Organization [WHO], 2020]. The disease is classified by diversification into the clinical course of the infection, ranging from a paucibacillary disease, in which few bacilli are present, to a multibacillary disease, in which a large bacillary load is present in lesions (Goulart et al., 2002). *M. leprae* is an obligatory intracellular bacillus and preferably infects macrophages and Schwann cells (Rambukkana, 2001).

Based on the natural history of the disease, it is observed that there is a form of partial resistance to *M. leprae* infection, tuberculoid leprosy (TL), in which the manifestations are related to the exacerbation of the cellular immune response (Th1), well-defined granuloma formation, limitation of lesions, and complete destruction of bacilli. At the other pole is the high susceptibility form, lepromatous leprosy (LL), which is characterized by a deficiency of cellular immune response and consequent polarization of the immune response to the humoral pattern (Th2), with excessive bacillary multiplication and dissemination of the disease for viscera and nervous tissue. It is a form of epidemiological importance because bacilli are massively present in skin lesions (Foss, 1997). Among these two polar forms are the unstable forms of the disease (dimorphic and indeterminate), with a broad spectrum of clinical manifestations, depending on the potentiality of the host cellular immune response to the parasite (Foss, 1997).

The susceptibility phenotype to *M. leprae* infection is complex and influenced by host and parasite factors, as well as environmental factors; however, some studies have suggested human genetic factors as being important in the acquisition of leprosy and the clinical course of the disease (Cardoso et al., 2010). Genetic changes can modify the transcription levels of a gene, and polymorphisms can occur not only in a protein-coding region (exon) but also in non-coding regions (intron and promoter region of the gene) and, therefore, influence the amount or composition of the protein produced by the gene (Santos et al., 2002; Moraes et al., 2004). Several genes have been associated with leprosy and are involved in susceptibility to leprosy in two different stages: leprosy *per se* and the development of different clinical forms (Sapkota et al., 2010; Silva et al., 2015; Tarique et al., 2015; Singh et al., 2018).

The purinergic receptor P2X7 (*P2RX7*) gene is located on the human chromosome 12 (q24O) at position 121,132,819–121,186,551 (GRCh38/hg38). This gene encodes the P2X7 receptor expressed in hematopoietic, mesenchymal, epithelial, and neural lineage cells. It plays a crucial role in immunity, inflammation, neurological function, bone homeostasis, and neoplasia (Wu et al., 2015). Activation of P2X7 by adenosine triphosphate (ATP) causes an immediate opening of a selective cation channel, allowing Ca^2+^ and Na^+^ influx and K^+^ efflux to occur. This process results in induction of the caspase cascade, apoptosis, and activation of phospholipase D. Phospholipase D promotes phagosome–lysosome fusion and causes mycobacterial death (Bahari et al., 2013; Yi et al., 2014).

Purinergic receptor P2X7 gene is highly polymorphic, with several single-nucleotide polymorphisms (SNPs) affecting the function of this receptor (Bahari et al., 2013). Several studies have reported *P2RX7* SNPs that resulted in loss or reduction of receptor function (Tekin et al., 2010). The SNP rs3751143 of the *P2RX7* gene is an exon polymorphism where the adenosine (A) changes to the cytosine (C). This exchange modifies the amino acid in the encoded protein (p.Glu496Ala) and generates a non-functional receptor (Gu et al., 2001). It was observed that monocytes from individual homozygous for the polymorphic C allele expressed non-functional receptors, whereas heterozygous individuals presented half of the *P2RX7* expression compared with functional protein (Gu et al., 2001). Functional loss polymorphisms lead not only to the reduction of P2X7 function but also to the impairment of ATP-induced mycobacterial death inside macrophages (Sun et al., 2010). This polymorphism was associated with susceptibility to tuberculosis in a cohort study of a Chinese population (Wu et al., 2015) and an Iran population (Amiri et al., 2018), in addition to other diseases, such as chronic Q fever (Jansen et al., 2019) and osteoporosis in postmenopausal women (Xu et al., 2017; Wang et al., 2018).

Another *P2RX7* polymorphism, rs1718119, confers function gain (Roger et al., 2010). It is a polymorphism where the guanosine (G) allele changes to the A allele. This exchange modifies the amino acid in the encoded protein (p.Thr348Ala). The G allele was associated with clinical signs of toxoplasmosis in a North American population. On the other hand, the allele A was associated with retinochoroiditis in a Brazilian population, where it was strongly protective (Jamieson et al., 2010). In a Chinese population, allele A was associated with a reduced risk for active tuberculosis (Zheng et al., 2017).

Early experiments showed that P2X7 activation potentiated killing of intracellular pathogens such as mycobacteria (Molloy et al., 1994), *Chlamydia* (Coutinho-Silva et al., 2003), *Toxoplasma* (Corrêa et al., 2010; Lees et al., 2010), and *Leishmania* (Chaves et al., 2009) mainly through facilitation of phagolysosome fusion and acceleration of acidification of parasitophorous vacuole, thus leading to the elimination of the microbial load (Morandini et al., 2014). A recent study by Salles et al. (2017) also demonstrated a key role of P2X7 in the response against the parasite *Plasmodium chabaudi*, showing that *P2RX7* null mice are more susceptible to malaria infection due to altered Th1 differentiation. Despite reports strongly suggesting that P2X7 is necessary for the development of cell-mediated acquired immunity for other infectious diseases (Corrêa et al., 2010; Lees et al., 2010; Salles et al., 2017), the type of immunity that is protective against *M. leprae*, there are no studies on the genetic association between leprosy and SNPs of *P2RX7*. In this work, we intend to fill this gap by investigating *P2RX7* polymorphisms and P2X7 messenger RNA expression levels (*in silico*) in Brazilian leprosy patients.

## Materials and Methods

### Subjects and Samples

Patients and healthy controls were recruited in Campos dos Goytacazes, Rio de Janeiro, southeast Brazil (21°45’15″S and 41°19’28″W, 13 m above sea level). A total of 334 subjects were included in this study: 171 (51.2%) patients and 163 (48.8%) healthy controls (Supplementary Table 1). Leprosy patients were grouped according to the WHO classification (Pardillo et al., 2007) in multibacillary (MB) or paucibacillary (PB) leprosy and Madrid classification (Davison et al., 1960) in LL, dimorph leprosy, indeterminate leprosy, and TL for the analysis, selected from Hansen Health Program from Campos dos Goytacazes Health Secretariat, which acts as a reference center. Healthy controls were unrelated individuals (volunteers) recruited from the local blood bank (hemocenter). All participants were clinically diagnosed according to the Brazilian’s Ministry of Health Guidelines, and the patient’s diagnosis was complemented with a bacilloscopy of suspected tissue lesions. Blood samples were collected by vacuum venipuncture in a tube containing citrate for genomic DNA extraction.

### Allele-Specific Expression Analysis

We used primary (unprocessed) RNA sequence data from the Sequence Read Archive public experiments. The biological samples included: primary healthy skin (Supplementary Dataset 1) (GEO BioProject PRJNA301173) from the study by Gupta et al. (2016) and skin patient lesions infected with *M. leprae* (Supplementary Dataset 1) from the study by Montoya et al. (2019) (GEO BioProject PRJNA518047). The second samples with lesions include de LL and TL. To estimate the extent and magnitude of allele-specific expression (ASE), we implemented PipASE (da Silva Francisco Junior et al., 2019) computational pipeline to identify, quantify, and sort out ASE sites in the transcriptome data. PipASE scans genome-wide for expressed single nucleotide variants in high-quality, aligned reads. They processed the RNA-seq data according to the best practice guidance using the ASEReadCounter tool from the open-source Genome Analysis Toolkit (GATK, v3.8), instrumented for variant discovery in high-throughput sequencing data (McKenna et al., 2010; DePristo et al., 2011; Van der Auwera et al., 2013). For the assessment of ASE, the read counts from the replicas were amalgamated, and Q1 values across each informative expressed single nucleotide variant site were calculated for all biosamples. The ASE across imputed heterozygous SNP sites was calculated as the difference of RNA-seq read counts between the two alleles, using the equation ASE = | 0.5 – Ref_allele_read count/(Ref_allele_read count + Alt_allele_read count)|. The allelic expression imbalance value per site (ranging between 0 and 0.5) is, therefore, a measure of departure from the expected Mendelian 1:1 allelic expression ratio. The ASE is then sorted like monoallelic expression, biallelic expression, or imbalance.

### Data Processing of Differentially Expression of Genes

Based on the finding of Montoya et al. (2019), we explored the differentially expressed genes (DEGs) in *M. leprae*-infected patients (GEO BioProject PRJNA518047) in comparison with healthy controls (GEO BioProject PRJNA301173). Significant values for DEGs in the three comparison groups, LL vs. Ctrl, TL vs. Ctrl, and TL vs. LL, were analyzed with DESeq2 (Love et al., 2014). The adjusted *p*-values were used to decrease the false-positive rate using Benjamini and Hochberg’s (BH) false discovery rate (FDR) method by default. Subsequently, log2(fold change) was calculated. An adjusted *p*-value < 0.05 was selected as a threshold value for DEG screening. The downregulated DEG signatures are modules of molecular identity to the infection success, as predicted by functional and literature enrichment analysis using gProfiler (Raudvere et al., 2019). The gene ontology analysis of unambiguous gene terms revealed DEG enrichment (significance threshold BH correction FDR *p* < 0.02) in cellular components and bioprocesses.

### Single-Nucleotide Polymorphism Selection

The selection of SNPs was based on the analysis of RNA-seq variants. After identifying the most frequent SNPs of the *P2RX7* gene, those associated with the infectious disease were selected. Another criterion for selecting the SNP was the minor allele frequency greater than 0.10 available in the dbSNP.^1^ For that, they were used as references as European populations (Central European University) and Africans (Yoruba in Ibadan, Nigeria) due to being the main genetic contributors of the population of the North of Rio de Janeiro. Full details of these SNPs are in Supplementary Table 2.

We interrogated the deleterious profiles for the SNPs shared between the two conditions (lesions and control). The variant pathogenicity was predicted using computational tools such as Sorting Intolerant from Tolerant (Ng and Henikoff, 2003) and Polymorphism Phenotyping (Adzhubei et al., 2013) according to the Ensembl Variant Effect Predictor database (McLaren et al., 2016).

### DNA Extraction

Genomic DNAs were prepared from blood samples using a commercial Illustra blood genomic Prep Mini Spin kit (GE Healthcare, Little Chalfont, United Kingdom) following the manufacturer’s instructions. Genomic DNA was quantified using a *NanoDrop* 2000c Lite Spectrophotometer (Thermo Fisher Scientific, Waltham, MA, United States) and kept frozen at −20 C until used. The genomic DNAs were used for multiplex polymerase chain reaction (PCR) experiments and SNP genotyping.

### Multiplex Polymerase Chain Reaction and Single-Nucleotide Polymorphism Genotyping

All oligonucleotide primers used in this study are listed in Supplementary Table 3. The SNP rs3751143 A>C and SNP rs1718119 G>A were genotyped by SNaPshot Kit (Thermo Fisher Scientific, Waltham, MA, United States). Amplification (multiplex PCR) was performed with the following concentration of reagents to a final volume of 12.5 μl: 2-mM magnesium chloride; 0.2-mM deoxyribonucleotide triphosphates, 15-mM Tris-hydrochloride, 50-mM potassium chloride; 10 pM of primer pair rs1718119, 40 pM of primer pair rs3751143, and 30 ng/μl of genomic DNA. Multiplex PCR was carried out using the following parameters: initial denaturation of 5 min at 95°C followed by 35 cycles of denaturation of 95°C for 30 s, annealing at 70°C for 30 s, extension at 72°C for 1 min, and a final polymerization at 72°C for 10 min. The reactions were processed in the GeneAmp^®^ 9700 or Veriti^®^ 96-Well VeriFlex thermocycler (Applied Biosystems).

The amplification products were purified using a mixture of 0.4 U of exonuclease I and 1 U of shrimp alkaline phosphatase. For each 3-μl aliquot of the amplified products, 1.5 μl of the enzyme mixture and 0.5 μl of the buffer solution were used. The reactions were carried out under the following conditions: 37 C for 1 h and 75°C for 15 min in the GeneAmp^®^ 9700 thermal cycler.

Sequencing by extension of a single nucleotide was performed using the SNaPshot multiplex kit (Applied Biosystems) in a final volume of 5 μl containing 2 μl of purified multiplex PCR product, 0.5 μl mixture of mini-sequencing primers (2 pM each), and 2.5 μl of SNaPshot multiplex ready reaction mix containing fluorescent dideoxynucleotides triphosphates. The reactions were carried out in the GeneAmp^®^ 9700 thermal cycler under the following conditions: 25 cycles of 96°C for 10 s, 50°C for 5 s, and 60°C for 30 s.

Single nucleotide extension sequencing products were purified using 0.3 U of the shrimp alkaline phosphatase enzyme. The reactions were carried out in the GeneAmp^®^ 9700 thermal cycler under the following conditions: 37°C for 30 min and 75°C for 15 min.

After purification, 0.54-μl aliquots of the products were added to a mixture of 9.45-μl formamide (Hi-Di Formamide) (Applied Biosystems) and 0.1 μl of GeneScan 120LIZ Size Standard (Applied Biosystems) and subjected to capillary electrophoresis using ABI 310 Applied Biosystems platform (Thermo Fisher Scientific, Waltham, MA, United States) calibrated with the Standard Set DS-02 Applied Biosystems Matrix.

The data analysis was performed using GeneScan^®^ Analysis and Genotyper^®^ software version 3.7 packages and GeneMapper^®^ ID version 3.2 (Applied Biosystems from Thermo Fisher Scientific, Waltham, MA, United States).

### Statistical Analysis

The Student and ^χ2^ tests were used to compare the age and sex among patients and control subjects, respectively. The genotype and allele frequencies were determined using Power Stat v.12. The ^χ2^ test was applied for each population (leprosy patients and control subjects) to investigate the Hardy–Weinberg equilibrium. ^χ2^ test was used for comparison of the genotype and allele frequencies, respectively, between leprosy patients and control subjects and also between the PB and MB patients. The odds ratio (OR) and *p*-values were calculated at GraphPad Prism 5.0 software, considering *p*-values less than 0.05 as significant.

## Results

### Differentially Expression Analysis

The genes that were consistently different from normal tissue (paired *t*-test, *p* < 0.05) were selected as DEG (Supplementary Table 4). As a result, 1,937 DEGs were chosen as genes that were upregulated in the *M. leprae*-infected tissue (log2FoldChange > 1 and *p*-value < 0.01), whereas 1,444 DEGs were chosen as genes that were downregulated (log2FoldChange < −1 and *p*-value < 0.01) (Supplementary Table 4). Among the upregulated genes were seven keratin-associated protein genes (KRTAP1-3, KRTAP3-2, KRTAP16-1, KRTAP19-1, KRTAP1-5, KRTAP5-8, and KRTAP5-7). The downregulated genes in *M. leprae*-infected tissue had cellular component relation with lysosomal membrane (*p*-value adj. 9.520590742324365e-21), lytic vacuole membrane (*p*-value adj. 9.520590742324365e-21), and lysosome (*p*-value adj. 7.675649093017353e-37), whereas the bioprocesses are related to immune system process (*p*-value adj. 4.05502263334811e-96), leukocyte mediated immunity (*p*-value adj. 8.673466507834307e-96) (Supplementary Table 5), indicating the interference of *M. leprae* in immune processes that leads to the establishment of the infection.

### Expression Profile of Purinergic Receptor P2X7 in *Mycobacterium leprae*-Infected Tissue

**FIGURE 1:**
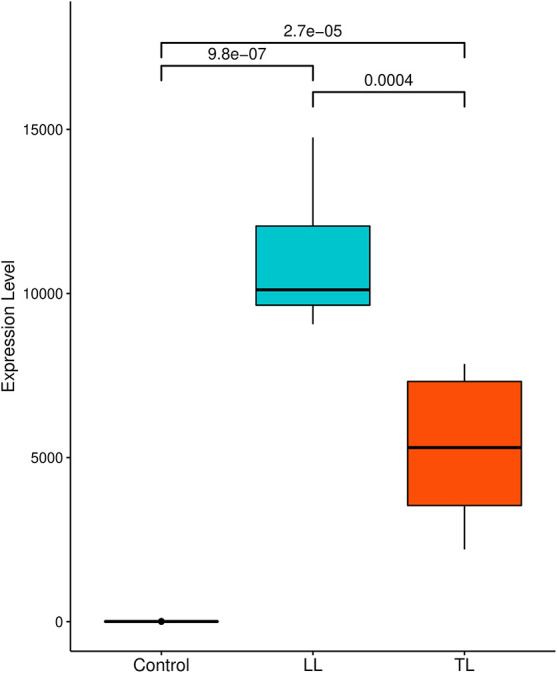
Comparison of P2X7 expression level under different conditions: box plot represents median and interquartile range of FPKM expression level of each sample. A significance test was performed to indicate whether expression level between three samples differed significantly. *X*-axes show disease conditions (LL and TL) and control; *Y*-axes are FPKM values. FPKM, fragments per kilobase per million mapped reads.

The expression of *P2RX7* calculated by the relative abundance of each transcript reported as fragments per kilobase per million mapped reads was more evident in the lesions than in the control samples. The comparison of conditions shows a significant difference between expression in the control and disease conditions with *p-*value < 9.8e-07 for LL and *p-*value < 2.7e-05 for TL. The expression between lesions is also evident with a higher level of expression for skin lesion LL with *p-*value = 0.0004 (Figure 1).

The *in silico* analysis using the PipASE identified 61 SNPs in the *P2RX7* gene. Of which, nine were expressed in lesions and control samples, 41 were expressed exclusively in control samples, and 11 are expressed exclusively in lesion samples. The more frequent SNPs identified in our analysis were rs1621388 (expressed in five patients with a skin lesion and three controls), rs1718119 (five patients with a skin lesion and seven control individuals), rs1718106 (four patients in skin lesion and eight individual controls), rs2230911 (only in a patient with lesion and four control individuals), rs7958311 (four patients with a skin lesion and seven control individuals), rs208294 (four patients in skin lesion and 10 control individuals), and SNP rs3751143 expressed in three patients with a skin lesion and one control individual (Supplementary Table 6). For SNP rs3751143, the expression profile of the patients (*n* = 3) presented monoallelic expression for the reference allele A, whereas, for the control (*n* = 1), the profile was monoallelic for the alternative allele C. This result is not to be compared with genotype data, until the C allele is a risk to leprosy development. For SNP rs1718119, all the control samples (*n* = 7) were monoallelic in the transcriptome for the alternative allele A, whereas in the skin lesion, the presence of both profiles [monoallelic for the alternative allele A (*n* = 2) and biallelic (*n* = 3)] in the patients for both lesions (Supplementary Table 7). This result can be associated with the genotype assay until the AA genotype was strongly associated with protection for leprosy development. A larger sample of transcriptome data is needed to allow a positive association between genotyping and expression profiles of rs3751143 and rs1718119. The pathogenicity prediction of the Ensembl Variant Effect Predictor database allowed us to identify six SNPs expressed by *P2RX7* in the control and lesion samples, predictably pathogenic in the Polymorphism Phenotyping and/or Sorting Intolerant from Tolerant database. For SNP rs3751143, the allele C was considered associated with the variant risk allele (Supplementary Table 7).

### Subjects and Samples

The average age differs between patients and controls (*p* < 0.0001). Of the patients, 110 are male, comprising 64.3% of the total value of individuals with the disease, whereas 61 are female, corresponding to 35.7%. According to the Madrid classification, the lepromatous and dimorphic clinical forms were most frequently diagnosed, with 35 and 39%, respectively (Table 1).

**TABLE 1:** Characteristics of the study population.

	**Patients (*n* = 171)**	**Controls (*n* = 163)**
**Age (years)**		
Mean	47.6 ± 19.3	36.2 ± 10.1
**Sex**	*n* (%)	*n* (%)
Male	110 (64.3)	105 (64.4)
Female	61 (35.7)	58 (35.6)

**Operational classification**	***n* (%)**	

MB	126 (74)	–
PB	45 (26)	–

**Madrid classification**	***n* (%)**	

Indeterminate	9 (5)	–
Tuberculoid	36 (21)	–
Lepromatous	60 (35)	–
Dimorphic	66 (39)	–

*PB, paucibacillary; MB, multibacillary; Student test for age between patients and control subjects, *p* < 0.0001; ^χ2^ test for sex between patients and control subjects, *p* = 1.*

Of the total samples collected from the recruited individuals, 297 of 334 were successful in the amplification and genotyping for the rs3751143, 140 in the case group and 157 in the control group. The 139 individuals in the case group were diagnosed in the clinical forms; one individual was not diagnosed in the clinical form. The 151 samples were genotyped for the rs1718119, 80 (53%) from the case group and 71 (47%) from the control group. One patient in the case group did not have the clinical form diagnosed. No deviations from Hardy–Weinberg equilibrium were observed in control groups in each polymorphism. In this study, assuming an alpha value of more than 0.05 was 0.07 for rs3751143 and 0.103 for rs1718119.

### Homozygosis of Purinergic Receptor P2X7 Gene rs3751143-C Allele Is a Risk Factor for Leprosy

The allele and genotype frequencies of the SNP rs3751143 in the *P2RX7* gene in leprosy patients and controls are shown in Table 2. No statistical difference was found in allele frequency between the case and control groups. However, in relation to the genotype frequency, it was observed that the CC genotype was strongly associated with leprosy development compared with the AC (*p* = 0.0271; OR = 2.686; CI = 1.183–6.096) and AA genotypes (*p* = 0.0274; OR = 2.42; CI = 1.140–5.138). This increased susceptibility was associated only with the C allele in homozygosis, seen in the recessive model (CC × AC + AA *p* = 0.0132; OR = 2.5; CI = 1.199–5.213), and no significance was observed considering the dominant model (CC + AC × AA).

**TABLE 2:** Allele and genotype distribution of polymorphisms rs3751143 in the *P2RX7* gene in patients with leprosy and controls.

	**Patients (140)**	**Control (157)**	**OR**	**95% CI**	***p***
C	83	71	1.442	0.9976–2.084	0.0605
A	197	243			
CC	24	12			
AC	35	47	2.686	1.183–6.096	**0.0271**
AA	81	98	2.42	1.140–5.138	**0.0274**
CC	24	12	2.5	1.199–5.213	**0.0132**
AC + AA	116	145			
CC + AC	59	59	1.21	0.7594–1.928	0.4763
AA	81	98			

*OR, Odds ratio; CI, confidence interval; ^χ2^ for comparison of genotype and allele frequencies between leprosy patients and control subjects and also between leprosy patients and controls subjects. The bold values represent statistical significance.*

Considering the WHO classification, no association was found in comparing genotypes and allele frequencies between MB and PB leprosy for polymorphisms rs3751143 A>C (Table 3).

**TABLE 3:** Allele and genotype distribution of polymorphisms rs3751143 in the *P2RX7* gene between multibacillary and paucibacillary leprosy.

	**MB (105)**	**PB (34)**	**OR**	**95% CI**	***p***
C	63	20	1.12	0.6138–2.044	0.7632
A	135	48			
CC	17	6			
AC	29	6	0.5025	0.1448–1.744	0.3438
AA	58	20	0.9623	0.3487–2.656	1
CC	17	7	0.7997	0.2995–2.135	0.6166
AC + AA	82	27			
CC + AC	46	13	1.402	0.6321–3.110	0.4309
AA	53	21			

*PB, paucibacillary; MB, multibacillary; OR, odds ratio; CI, confidence interval; ^χ2^ for comparison of genotype and allele frequencies between the PB and MB patients.*

### rs1718119-A Allele of the Purinergic Receptor P2X7 Gene Is a Protective Factor for the Development of Leprosy

The allele and genotype frequencies of the SNP rs1718119 in the *P2RX7* gene in leprosy patients and controls are shown in Table 4. Statistical difference was found in allele frequency between the case and control groups. Data show that there is a protection factor associated with the polymorphic allele A (*p* = 0.0014; OR = −0.4154; CI = 0.2430–0.7100). In relation to the genotype frequency, it can be observed that the AA genotype was strongly associated with protection for leprosy development compared with the GG (*p* = 0.034; OR = 0.2540; CI = 0.07235–0.8914) but were not compared with the AG genotypes (*p* = 0.7502; OR = 0.6667; CI = 0.1804–2.463). This protective factor is associated with the presence of allele A, as evidenced when we consider the dominant model (AA + AG × GG *p* = 0.0028; OR = 0.03516; CI = *0.0*.1801–0.6864) but not with the recessive model (AA × AG + GG).

**TABLE 4:** Allele and genotype distribution of polymorphisms rs1718119 in the *P2RX7* gene in patients with leprosy and controls.

	**Patients (80)**	**Control (71)**	**OR**	**95% CI**	***p***
A	28	48	0.4154	0.2430–0.7100	**0.0014**
G	132	94			
AA	4	9			
AG	20	30	0.6667	0.1804–2.463	0.7502
GG	56	32	0.2540	0.07235–0.8914	**0.034**
AA	4	9	0.3626	0.1065–1.234	0.1446
AG + GG	76	62			
AA + AG	24	39	0.3516	0.1801–0.6864	**0.0028**
GG	56	32			

*OR, odds ratio; CI, confidence interval; ^χ2^ for comparison of genotype and allele frequencies between leprosy patients and control subjects and also between leprosy patients and controls subjects. The bold values represent statistical significance.*

Considering the WHO classification, no association was found in comparing genotypes and allele frequencies between MB and PB leprosy for polymorphisms rs1718119 G>A (Table 5).

**TABLE 5:** Allele and genotype distribution of polymorphisms rs1718119 in the *P2RX7* gene between multibacillary and paucibacillary leprosy.

	**MB (60)**	**PB (19)**	**OR**	**95% CI**	***p***
A	20	8	0.75	0.3001–1.875	0.6262
G	100	30			
AA	4	0			
AG	12	8	3.462	0.3437–34.86	0.3746
GG	44	11	1.333	0.1419–12.52	1.0
AA	4	0	1.754	0.1930–15.95	1.0
AG + GG	56	19			
AA + AG	16	8	0.5	0.1705–1.466	0.2548
GG	44	11			

*PB, paucibacillary; MB, multibacillary; OR, odds ratio; CI, confidence interval; ^χ2^ for comparison of the genotype and allele frequencies between the PB and MB patients.*

## Discussion

Genetic studies of the association are fundamental to elucidate the mechanisms inherent in diseases in general, whether infectious or not. The occurrence of these diseases is controlled by a genetic component associated with environmental, socioeconomic, and cultural factors, among others, resulting in integrated actions that might modify the expression of a varied number of genes. Montoya et al. (2019) have established that the transcriptional profiles of some immune genes linked to antibody expression in infected patients may reflect the variation of clinical disease manifestations (Montoya et al., 2019). Based on their finding, we further explored the DEGs in *M. leprae-*infected patients, which differed from healthy controls. One of the genes that have its expression modified is the *P2RX7*, present in studies of RNA-seq with higher expression level in lesion tissue than in control samples (Figure 1); this evidence indicates that the infection modulates the expression of various kinds of genes. The gene *P2RX7* was considerably pathogenic and may be related to gain or loss of pathogenicity, according to Landrum et al. (2016) and Kaminsky et al. (2011) analyzed in integration with the ClinVar and ClinGen CNV bowsers.

*In silico* analysis of variants using studies of RNA-seq, the polymorphism rs1718119 was seen in patients and control; however, all the control samples were monoallelic in the transcriptome for the alternative allele A. These data are similar to this study, where individuals with allele A were less likely to develop leprosy. On the other hand, the polymorphism rs3751143 showed contrasting results, with the allele C in monoallelic status in control individuals and allele A (monoallelic) in patients with leprosy. However, some factors need to be considered with this result. The analysis considered the transcriptome, not the genotype, as shown in *in vitro* analysis in this study, where the allele of risk was related with leprosy patients. In addition, a limited number of patients (three) and control (one) were found in *in silico* studies, whereas more than 100 were tested *in vitro* for genotype, which demonstrates that the results obtained *in silico* are not significant. To corroborate the *in vitro* analysis, the browser for risk variants was consulted, showing the allele of the risk C in rs3751143.

Association studies are based on comparing the allelic frequencies of a genetic marker between affected and unaffected individuals. Certain alleles are considered to be associated with the phenotype studied when it occurs with a different frequency between affected individuals compared with control individuals. In the context of leprosy, some immune response genes have already been investigated in genetic association studies, such as *IL18R1*, a gene that encodes the cytokine receptor interleukin (IL)-18, which has been associated as a risk factor for leprosy in a Chinese population (Liu et al., 2012). IL-18 can promote Th1 responses to *M. leprae*. Also, in the Th1 profile, the *TNFA* gene rs1800629 polymorphism was studied in leprosy in several ethnic groups, and through a meta-analysis, this polymorphism was associated with a protective effect against the risk of leprosy in the Latin American population (Areeshi et al., 2017). This is the first study to associate the genetic variants c.1513A>C and c.1068G>A of *P2RX7* with leprosy. The presence of the CC genotype of the rs3751143 polymorphism is associated with an increase, about twice as much, of susceptibility to the development of leprosy compared with the presence of the AC and AA genotypes. Increased susceptibility associated with the C allele was shown to be recessive. These data corroborate the findings regarding other intracellular pathogens, such as *Mycobacterium tuberculosis*. In this sense, a case–control study demonstrated that the rs3751143-C allele increases the susceptibility to extrapulmonary tuberculosis (Fernando et al., 2007; Nino-Moreno et al., 2007; Ben-Selma et al., 2011). In addition, ATP-mediated mycobacterial death was ablated in macrophages of homozygous individuals for the rs3751143-C allele and significantly decreased bacterial replication inside macrophages of heterozygous individuals (Fernando et al., 2007). Similarly, macrophages from individuals with the rs3751143-C polymorphism are less effective in killing intracellular *Toxoplasma gondii* after exposure to ATP compared with macrophages of persons with reference allele rs3751143-A. Supporting a specific effect of P2X7 on *T. gondii*, macrophages from *P2RX7* knockout mice are unable to kill *T. gondii* as effectively as wild-type mouse macrophages (Lees et al., 2010). This polymorphism was also associated with the development of chronic Q fever, which is a persistent infection, mostly of aortic aneurysms, vascular prostheses, or damaged heart valves, caused by the intracellular bacterium *Coxiella burnetii* (Jansen et al., 2019). Regarding the rs1718119 polymorphism, we found a protective association between the polymorphic allele and the development of leprosy. This same allele also provided protection in other studies with infectious diseases, such as toxoplasmosis (Jamieson et al., 2010) and tuberculosis (Zheng et al., 2017).

One polymorphism of loss of function and another of gain of function of the *P2RX7* gene, associated with susceptibility and protection, respectively, both found here in this study, suggests the involvement of P2X7 in the leprosy immunopathology. The loss of function polymorphism is located in the cytoplasmic tail of the carboxyl-terminal (Gu et al., 2001). This region of the receptor is involved in the formation of pores induced by ATP, one of the functional properties of the receptor (Surprenant et al., 1996); it is known that the formation of pores in the cell membrane *via* P2X7/ATP is involved in cell lysis; therefore, the alteration of this receptor functionality on the surface of macrophages, host cell of *M. leprae*, may influence the response to the bacillus. The genetic study associating the loss of function polymorphism and leprosy showed susceptibility to infection, demonstrating that the resistance to the development of the disease is impaired, and we hypothesize that the polymorphism may influence the immune response to *M. leprae*. Thus, like the function loss polymorphism, the function gain polymorphisms also influence the response *via* the receptor. The function gain polymorphism studied here is present in the transmembrane domain 2 of the receptor, close to the region believed to control the permeability pathway (Stokes et al., 2010). Ursu et al. (2014) observed that rs1718119 was expressed at higher levels in transfected HEK 293 cells, as well as rs3751143 and another loss of function polymorphism showed lower levels than the control, but they did not observe an effect different from the gain polymorphism function in relation to the functions of P2X7, pore formation, and channel opening. Stokes et al. (2010) observed a higher secretion of IL-1β from monocytes of homozygous individuals for the function gain polymorphism, and the secretion was completely decreased when using a P2X7 antagonist. In this sense, P2X7 is considered one of the most potent activators of the NLR family pyrin domain containing three inflammasome, where extracellular ATP is a strong stimulus for the release of IL-1β, through the K^+^ efflux in caspase-1 activation and IL-1β processing (Grassi, 2020). Extracellular ATP can induce the bactericidal activity of macrophages toward mycobacteria. Macrophages produce various chemokines (RANTES and MCP-1) and cytokines (TNF-α and IL-1β) after mycobacterial infection, and these effector immune molecules are necessary for the recruitment and activation of leukocytes and the subsequent control of mycobacterial infection (Flynn and Chan, 2001) and other intracellular pathogens (Grassi, 2020). The control of infection in the context of leprosy is seen in PB clinical forms, considered a profile of resistance to infection. Although we did not observe an association between the rs1718119 polymorphism in the severity of the disease, a protective factor was observed in the development of the disease *per se*. It is not possible to determine whether the polymorphism and the effect caused on the receptor are involved in the response generated in the infection by *M. leprae*; therefore, further studies are necessary, as well as for the interaction with loss of function polymorphisms.

**FIGURE 2:**
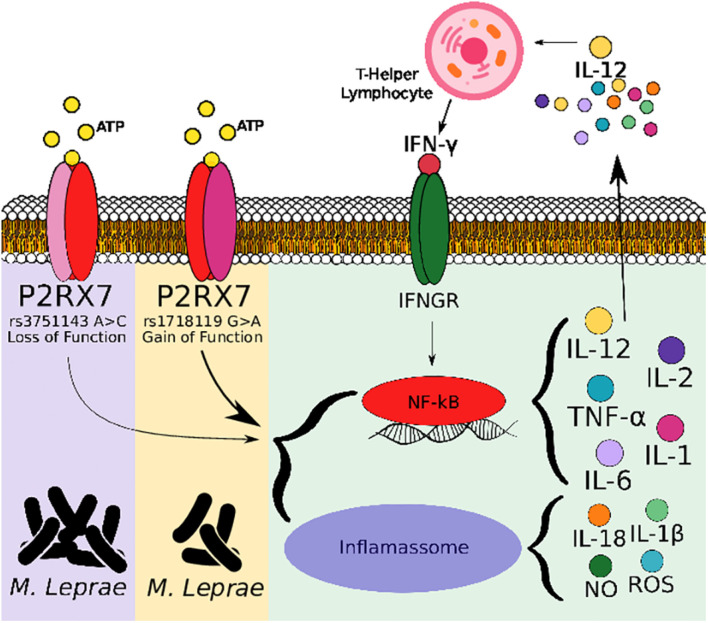
Schematic hypothesis of *Mycobacterium leprae* elimination following ATP ligation of P2X7. *M. leprae* and extracellular ATP released during infection triggers P2X7 signaling cascade, which culminates in nuclear factor-kappa B translocation to nucleus, stimulating secretion of cytokines and chemokines, inducing recruitment of inflammatory cells and producing inflammatory mediators such as reactive oxygen species and nitric oxide, which can either direct stimulate mycobacteria elimination or activate NLR family pyrin domain containing three inflammasome for this elimination. P2X7 receptor’s loss-of-function polymorphism (rs3751143) have been linked to impaired capacity of macrophages to eliminate bacillus contrary to P2X7 receptor’s gain-of-function polymorphism (rs1718119).

In addition, our findings in the *in silico* study show increased P2X7 expression in individuals with the tuberculoid and lepromatous clinical forms compared with the control. Similarly, our association data suggest functional P2X7 seems to be important for an effective immune response against *M. leprae*. The involvement of P2X7 in the immunopathology of leprosy is not yet known; however, P2X7 exerts important regulation in the infection caused by *M. tuberculosis* and other intracellular pathogens, suggesting that anti-mycobacterial mechanisms can be genetically regulated for this receptor in *M. leprae* too (Figure 2). In addition, P2X7 is also involved in differentiating T cells to a Th1 profile, where Salles et al. (2017) demonstrated that P2X7 promotes differentiation of Th1 instead of Thf in the infection with *P. chabaudi*, a protozoan that causes malaria in rodent mammals. *P2RX7* knockout mice were more susceptible to infection and had an impaired Th1 response differentiation. In the *in vitro* assay, treatment with a P2X7 antagonist inhibited the proliferation of TCD4 cells and the production of interferon-gamma (IFN-γ), confirming the involvement of ATP and P2X7 (Salles et al., 2017). An inadequate induction of the Th1/Th2 response differentiation determines a clinical manifestation of resistance or susceptibility. IFN-γ is a crucial cytokine for protection against mycobacteria; it is known that an effective cellular immune response is important in the clinical manifestation of resistance in leprosy. Upadhyay et al. (2019) demonstrated a higher production of IFN-γ in the peripheral blood mononuclear cell culture supernatant of patients with the tuberculoid clinical form than in patients with the lepromatous form after stimulation with *M. leprae* antigens. P2X7 is a receptor that is part of innate immunity; this fact highlights the findings of genetic association, considering that the resulting association was observed in the manifestation of the disease and not in severity when we correlated as MB and PB forms, as is seen in this study. In addition, the degree of disability, another parameter of severity, was not associated with the different genotypes of the studied polymorphisms (data not shown).

Our study brings new information about P2X7 in the context of leprosy in the studied population; however, other genetic studies in different populations are needed to predict these polymorphisms as genetic markers for leprosy. Immunological studies are also needed to determine the involvement of this receptor and its expression modified by polymorphisms present in the P2RX7 gene in the death of *M. leprae*.

## Data Availability Statement

The datasets presented in this study can be found in online repositories. The names of the repository/repositories and accession number(s) can be found below: https://www.ncbi.nlm.nih.gov/SNP/snp_ss.cgi?ss=5314585658 and https://www.ncbi.nlm.nih.gov/SNP/snp_ss.cgi?ss=2137544277.

## Ethics Statement

The studies involving human participants were reviewed and approved by the Faculdade de Medicina de Campos/Fundação Benedito Pereira Nunes. Written informed consent to participate in this study was provided by the participants’ legal guardian/next of kin.

## Author Contributions

RS performed the data collection, experimental assays, data analyses, and manuscript preparation. TL performed the experimental tests and data analyses. CF performed *in silico* analyses and writing. LN performed the data collection and sample processing. EN performed the clinical diagnostics. AP-R performed the study design and manuscript preparation. All authors contributed to the article and approved the submitted version.

## Conflict of Interest

The authors declare that the research was conducted in the absence of any commercial or financial relationships that could be construed as a potential conflict of interest.

## Publisher’s Note

All claims expressed in this article are solely those of the authors and do not necessarily represent those of their affiliated organizations, or those of the publisher, the editors and the reviewers. Any product that may be evaluated in this article, or claim that may be made by its manufacturer, is not guaranteed or endorsed by the publisher.

## Footnotes

1
https://www.ncbi.nlm.nih.gov/snp/

## Supplementary Material

Supplementary Table 1 Genotyped samples.

Supplementary Table 2 Details of the genotyped SNPs in this study.

Supplementary Table 3 List of oligonucleotides used in this study.

Supplementary Table 4 Differentially expressed genes.

Supplementary Table 5 Functional Analysis of Gene Ontology for DEG genes.

Supplementary Table 6 SNPs for P2RX7.

Supplementary Table 7 Clinical variant information.

Supplementary Dataset 1 Samples.

## Abbreviations

ASE: allele-specific expression
ATP: adenosine triphosphate
DEG: differentially expressed genes
LL: lepromatous leprosy
MB: multibacillary
*P2RX7*: purinergic receptor P2X7 gene
P2X7: purinergic receptor P2X7 protein
PB: paucibacillary
SNP: single-nucleotide polymorphism
T*h*1: T *helper* 1
T*h*2: T *helper* 2
TL: tuberculoid leprosy.
